# Cardiopulmonary responses to maximal aerobic exercise in patients with cystic fibrosis

**DOI:** 10.1371/journal.pone.0211219

**Published:** 2019-02-13

**Authors:** Craig A. Williams, Kyle C. A. Wedgwood, Hossein Mohammadi, Katie Prouse, Owen W. Tomlinson, Krasimira Tsaneva-Atanasova

**Affiliations:** 1 Children’s Health and Exercise Research Centre, Sport and Health Sciences, University of Exeter, Exeter, United Kingdom; 2 Department of Mathematics and Living Systems Institute, University of Exeter, Exeter, United Kingdom; 3 Centre for Biomedical Modelling and Analysis, University of Exeter, Exeter, United Kingdom; 4 EPSRC Centre for Predictive Modelling in Healthcare, University of Exeter, Exeter, United Kingdom; Sao Paulo State University—UNESP, BRAZIL

## Abstract

Cystic fibrosis (CF) is a debilitating chronic condition, which requires complex and expensive disease management. Exercise has now been recognised as a critical factor in improving health and quality of life in patients with CF. Hence, cardiopulmonary exercise testing (CPET) is used to determine aerobic fitness of young patients as part of the clinical management of CF. However, at present there is a lack of conclusive evidence for one limiting system of aerobic fitness for CF patients at individual patient level. Here, we perform detailed data analysis that allows us to identify important systems-level factors that affect aerobic fitness. We use patients’ data and principal component analysis to confirm the dependence of CPET performance on variables associated with ventilation and metabolic rates of oxygen consumption. We find that the time at which participants cross the gas exchange threshold (GET) is well correlated with their overall performance. Furthermore, we propose a predictive modelling framework that captures the relationship between ventilatory dynamics, lung capacity and function and performance in CPET within a group of children and adolescents with CF. Specifically, we show that using Gaussian processes (GP) we can predict GET at the individual patient level with reasonable accuracy given the small sample size of the available group of patients. We conclude by presenting an example and future perspectives for improving and extending the proposed framework. The modelling and analysis have the potential to pave the way to designing personalised exercise programmes that are tailored to specific individual needs relative to patient’s treatment therapies.

## Introduction

Cystic fibrosis (CF) is the most common life shortening genetic disease in the Caucasian population, affecting nearly 11,000 individuals in the United Kingdom (UK) [1] and predominantly manifests itself throughout the respiratory, digestive and reproductive systems of the human body. The genetic mutation responsible for CF results in reduced trans-epithelial chloride transport, and increased sodium and water absorption, thus reducing the hydration status of the mucosal lining of the airway and digestive systems. The resultant viscous mucus is liable to increased infection and further inflammation and a progressive decline in lung function [2]. Currently there is no cure for CF and therefore CF is a diseased that is managed with appropriate medication, nutrition, physiotherapy and exercise. Enhanced aerobic fitness as represented by maximal oxygen consumption (${\dot{\text{V}}\text{O}}_{2\text{max}}$) is associated with lower risk of hospitalisation, increased exercise tolerance, reduced residual volume, increased endurance of the respiratory muscles, enhanced sputum expectoration and decreased rate of decline in pulmonary function [3–7]. Furthermore, individuals with CF possessing a higher ${\dot{\text{V}}\text{O}}_{2\text{max}}$ are shown to have a reduced mortality risk, as Nixon et al. [8] reported that adults with a ${\dot{\text{V}}\text{O}}_{2\text{max}}$ greater than 82% of their predicted value had an 83% 8-year survival rate, compared to just 28% 8-year survival rate for patients with a ${\dot{\text{V}}\text{O}}_{2\text{max}}$ less than 58% of their predicted value [8]. This enhanced survival has also been reported in a paediatric cohort, with a 100% survival rate after 7 years in children with CF with a ${\dot{\text{V}}\text{O}}_{2\text{peak}}$ over 45 ml^.^kg^-1.^min^-1^ [9].

Given the importance of identifying and monitoring aerobic fitness, cardiopulmonary exercise testing (CPET) is recommended to take place on at least an annual basis [10], to provide a clinically useful prognostic evaluation of a patient’s functional capabilities. Even in mild to moderate severity of CF, patients are known to demonstrate impairments in cardiac and respiratory functions leading to exercise intolerance which are responsive to exercise training, and can result in an enhanced quality of life, increased physical function and increased life expectancy [11, 12].

Effective management of the disease is of critical importance due to an aging CF population group (median predicted survival of children born with CF in the UK is currently 45 years [1]) and high medical care costs [13]. Exercise is widely acknowledged as a key management strategy for CF, supported by mechanistic data on the systemic effects of exercise at the cellular level *in vivo* in young patients with CF [6–8, 11, 14]. However, an integrated systems level understanding of the limitations of aerobic fitness for CF patients is lacking. Measurement techniques that do exist to quantify within-organ, real-time perfusion and intracellular oxygenation are invasive and unethical for use with paediatric patients, and current animal model research provides limited direct relevance to paediatric pathology. In clinical practice, there is significant interaction between cardiac, pulmonary and musculoskeletal function, which can result in the functional improvement in one part of the combined system, but detrimental effects on others [11]. Clinicians therefore inevitably have to adopt very imprecise guidelines related to exercise prescription [15].

The use of modelling and simulation tools in clinical medicine is currently the subject of intense research interest both in the UK and internationally [16–20], and the adoption of a systems biomedicine approach to build and validate novel multi-scale, organ-level, integrated, re-usable and re-deployable models represents a paradigm shift in biomedical modelling and simulation. There are numerous organ level models in existence [21–24], however, to date there have been limited attempts to either integrate these or to apply them to real clinical applications. There is ongoing basic science and clinical trial work providing data on the micro [25] and macrovascular [26] changes associated with exercise. These data, although important, have yet to be integrated quantitatively with other data streams. In particular, there has been no previous work on the use of predictive modelling and simulation technologies for developing treatment strategies for CF patients.

Therefore the aim of this study is twofold. Firstly, to analyse the physiological responses to progressive exercise in patients with CF, with a view of determining predictors of performance. Secondly, to develop a surrogate (statistical) model that allows the evaluation of how CF impairs exercise tolerance relative to increasing ventilatory and metabolic demands. Specifically, how various ventilatory parameters quantitatively affect the gas exchange threshold (GET) for a group of patients is explored.

## Materials and methods

This study was a retrospective analysis of existing CPET data from 15 children and adolescents with CF. Original ethics approvals was approved by South West NHS Research Ethics Committees [10/H0107/78; 13/SW/0166; 14/SW/0061] and written informed consent and assent was obtained from parent(s)/guardian(s) and participants, respectively in accordance with the Declaration of Helsinki.

### CPET data

As part of original studies, all participants performed a valid [27] and reliable [28] combined ramp incremental and supramaximal (S_max_) CPET to determine ${\dot{\text{V}}\text{O}}_{2\text{max}}$ and the GET. This protocol was performed on an electronically braked cycle ergometer, and required patients to perform an initial exhaustive ramp incremental test at a pre-determined rate between 10–25 W∙min^-1^, in order to elicit exhaustion in approximately ten minutes [29]. After a 3-min warm-up at 10–20 W, participants completed this incremental test to the point of volitional exhaustion, maintaining a cadence of 70–80 rpm throughout. Exhaustion was defined as a 10 rpm drop in cadence for five consecutive seconds, despite strong verbal encouragement. Active (5-min cycling at 20 W) and then passive seated recovery (10 min) then preceded the S_max_ bout. S_max_ verification consisted of a 3-min warm-up (10–20 W), followed by a ‘‘step” transition to a constant work rate corresponding to 110% peak power output [30] obtained during the ramp incremental phase.. Upon volitional exhaustion (defined previously), a 5-min active recovery (slow cycling at 20 W) concluded the combined CPET session. ${\dot{\text{V}}\text{O}}_{2\text{max}}$ and the GET were subsequently identified for all participants using established methods which are validated for use in CF [31].

### Models and simulations

Simulations are widely used in various fields of science and engineering because conducting physical experiments is too costly, or highly time-consuming, or even impossible in some cases [32]. In the case of CPET in CF patients, there are also ethical considerations, since the test adds to the treatment burden many children and adolescents with CF already face.

Often, a primary goal of using model simulations is to perform quantitative studies such as uncertainty quantification or sensitivity analysis. Such studies are crucially important in biomedicine, since there exists significant variation both between and within patient groups. Through understanding and quantification of the uncertainty within the mathematical models, outcomes of patient-specific interventions can be better predicted. However, such investigations require a large number of runs that makes it impractical if each run takes more than a few seconds. To cope with this difficulty, one can use *emulators*, also known as *surrogates*, or *metamodels* or *response surfaces* [33]. These provide a fast approximation of the input/output relation governed by the underlying simulator. The most important classes of surrogate models have been described elsewhere [34–36].

The surrogate model employed in this study is based on Gaussian processes (GPs), which have become increasingly popular over the last two decades [33]. A Gaussian Process (GP) defines a probability distribution over functions where the true function is considered as a particular sample path. GPs have been used in a wide range of applications from wireless communication, to obtain position estimates for a mobile user [37]; metallurgy, to model the development of microstructure [38]; and in biology, to describe gene regulatory processes and cell growth [39–41]. A GP defines a probability distribution over functions which is fully specified by its mean, μ, and covariance *K* [42], which are both functions of the input variables: μ = μ(**x**), *K* = *K*(**x,x’**). While μ(**x**), could be any function (though in practice is often chosen to have a polynomial dependence on **x)**, the covariance function is required to be positive semi-definite [42]. Specifically, if *Y* is a GP defined on the space of input variables denoted by *D*, then we write:

$$Y∼GP(\mu ,K):\;\mu (x)\;=\;E[Y(x)],\;K(x,x^{\prime })\;=\;Cov(Y(x),Y(x^{\prime })),\;\forall \;x,x^{\prime }\in D\subset R^{d}$$

**Fig 1:**
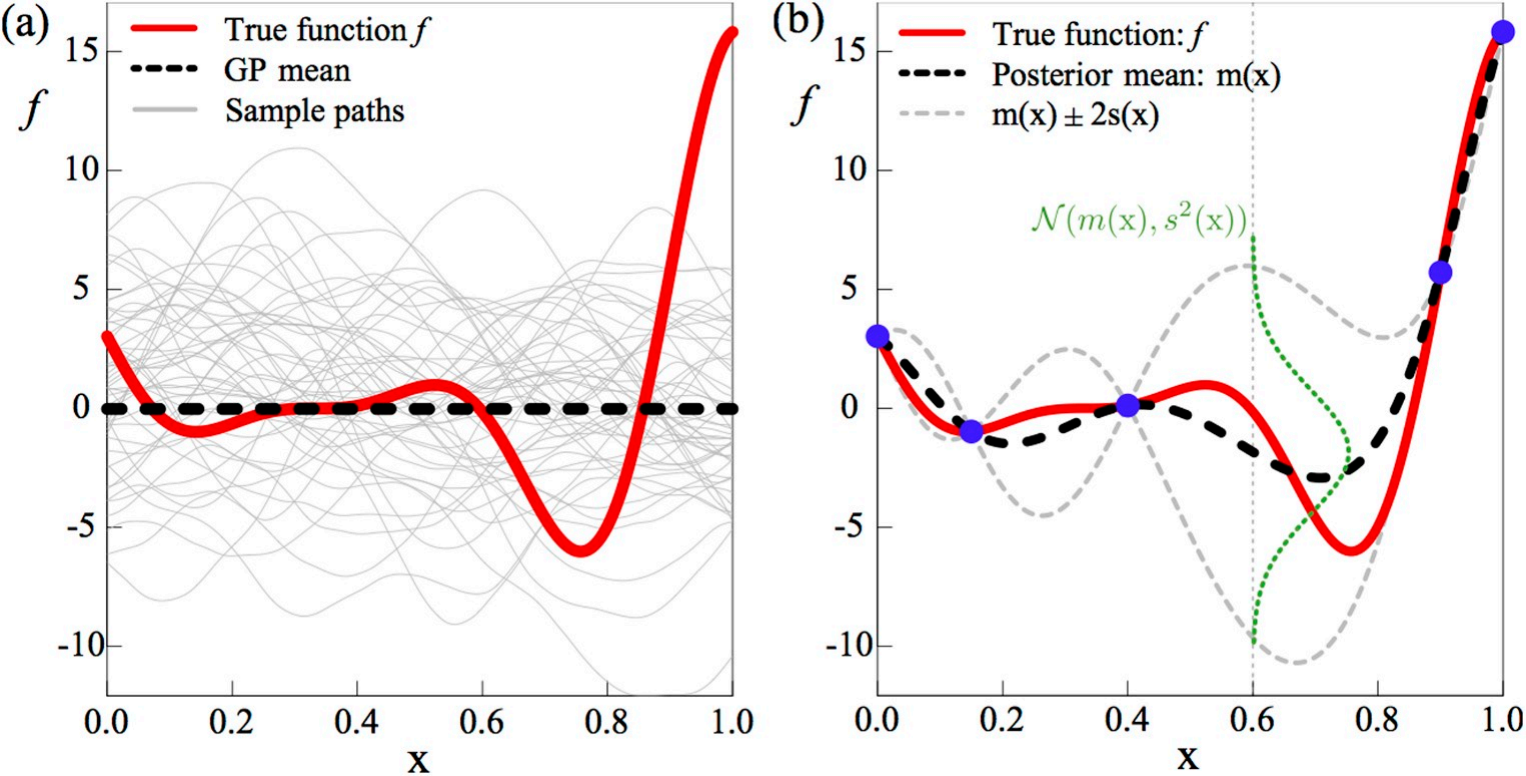
GP is a probability distribution over functions. The thick solid red line is the true function *f* and the thick dashed black line is the GP “prior”. (a) Thin grey lines show sample functions of the GP. (b) Blue bullets indicate five data points sampled from *f*. The GP distribution is updated using these sample data points. The thin grey dashed lines show *m*(x) ± 2*s*(x).

The above can be regarded as “prior” distribution over function spaces. This can be seen more clearly in Fig 1(a). In this subfigure, which shows a generic example of a GP, the bold red line is the ‘true’ function *f*. Note that the true function is unknown—our aim is to construct a model that approximates it. The thin grey lines are sample functions of a GP distribution with μ(**x**) = 0 and a squared-exponential covariance function given by:

$$K(x,x^{\prime })\;=\;\sigma^{2}\prod_{i\;=\;1}^{d}exp(-\frac{{\left|x_{i}-x_{i}^{\prime }\right|}^{2}}{2\Phi_{i}^{2}}),$$

Which is a popular choice in GP modelling. Here, σ controls vertical variability of *sample functions* and *Φ*_*i*_ > 0, *i* = 1, …, *d*, governs the degree of smoothness of them along the input dimension *i*. These parameters are usually unknown and estimated via maximum likelihood method [42]. For exposition purposes, the example plot is restricted to the case *d =* 1, but the approach is unchanged for *d* > 1.

Thus far, we have defined the prior distribution for the GP. It is clear from Fig 1(a) that, in general, prior distributions are unlikely to provide a good approximation to the true function *f*. We can compute the GP “posterior” distribution by incorporating data points (known as “training data”) obtained from evaluating *f* at specific points, following a Bayesian framework. The resulting posterior distribution of the GP, conditioned on the data, will be much closer to the true function.

Let **y** = {*f*(**x**^1^), …, *f*(**x**^*n*^)} be a set of function evaluations (or observations) at *n* locations **X** = {**x**^1^, …, **x**^*n*^}. Here, function evaluations correspond to the GET location for a set of patients during the CPET. Predicting with GP is obtained by conditioning it Y on sample points the observations, i.e. Ω = {X, y} Y(x)|Y(x^1^) = f(x^1^),…,Y(x^n^) = f(x^n^). For any (new) z ∈ D, this conditional distribution has a normal distribution with the mean m(z) and variance s^2^(z) which are used as the prediction and the associated uncertainty at *z* posterior, respectively. They are expressed by distribution of Y(z)|Ω has a normal distribution with the following mean, *m*, and variance, *s*^*2*^:

$$m(z)\;=\;\mu (z)+K{\left(z,X\right)}^{T}K{\left(X,X\right)}^{-1}(y-\mu )$$ (1)

$$s^{2}(z)\;=\;K(z,z)-K{\left(z,X\right)}^{T}K{\left(X,X\right)}^{-1}K(z,X)$$ (2)

where ^T^ denotes the transpose operator, ^-1^ is the inverse operator and **μ =** μ**(X)** is the vector of the mean function at **X**. In addition, *K*(**z,X**) and *K*(**X,X**) are the covariance vector between *Y*(**X**) and Y(**z**) and the covariance matrix between the observations. Fig 1(b) shows an example of incorporating sample points to update the prior distribution shown in Fig 1(a). In this generic example, the function *f* is evaluated at five distinct values of **x,** and the mean and variance of the GP are updated using (1)-(2).

At the evaluated points, indicated in blue (colour online), the true value of *f* is known and so the variance of the GP at these points vanishes and m(**x**) = *f(***x***)*. In between these points, the variance increases, dependent on the distance (in terms of **x)** from a sampled point. The mean of the GP, shown by the thick black dashed line now approximates the true function much more closely (recall that the prior mean function in Fig 1(a) was zero everywhere), and matches exactly at the evaluated points. The approximation can be further improved by incorporating more data points (function evaluations), particularly around those input values for which the prediction variance *s*^*2*^*(****x****)* is high.

The data analysis was performed using Python (Anaconda Software Distribution. Version 2–2.4.0. Continuum Analytics, 2016. URL https://continuum.io) and MATLAB and Statistics Toolbox Release 2016b, The MathWorks, Inc., Natick, Massachusetts, United States. The GP model simulator was implemented in R (R Core Team (2013). R: A language and environment for statistical computing. R Foundation for Statistical Computing, Vienna, Austria. URL http://www.R-project.org/.)

## Results

### Data analysis

**Fig 2:**
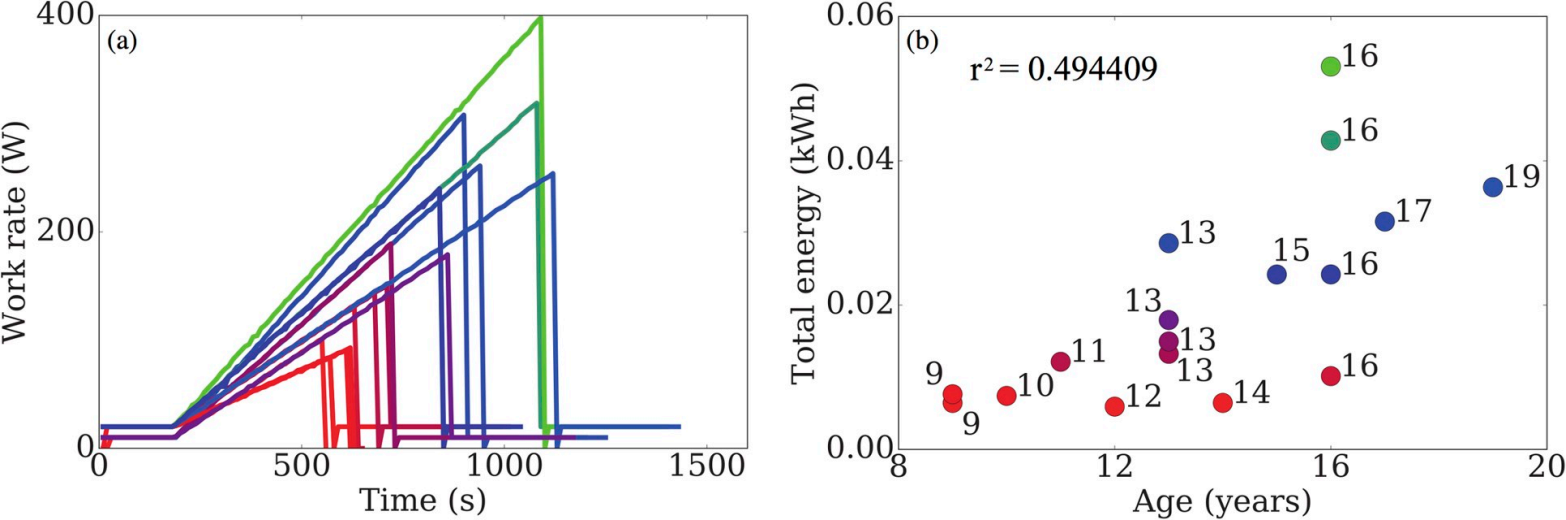
(a) The work rate for each participant is increased at a rate dependent on their past test performance. (b) Participant age is correlated with test performance for young participants, but not for older ones.

To facilitate understanding, we first plot in Fig 2(a), raw data displaying the performance of the participants. The work rate for each participant is increased at a rate that is either, a) dependent on their performance in previous tests, or b) when a prior test is unavailable, at a rate that is predicted to elicit exhaustion in approximately ten minutes [29]. This is done in order to keep the expected duration of the test comparable to other participants. Note that this means that the total energy expended by a given participant is not based on the duration of the test alone. In Fig 2(b), we show how participant age affects overall test performance. We observe a correlation between the two: the worst performing participants tend to be the youngest, but this effect is insignificant at older ages. The colour coordination used in this figure (red-worst performance → blue-average performance →green-best performance) will be used throughout the remainder of this section, where performance is quantified by the total energy transferred during the test.

**Fig 3:**
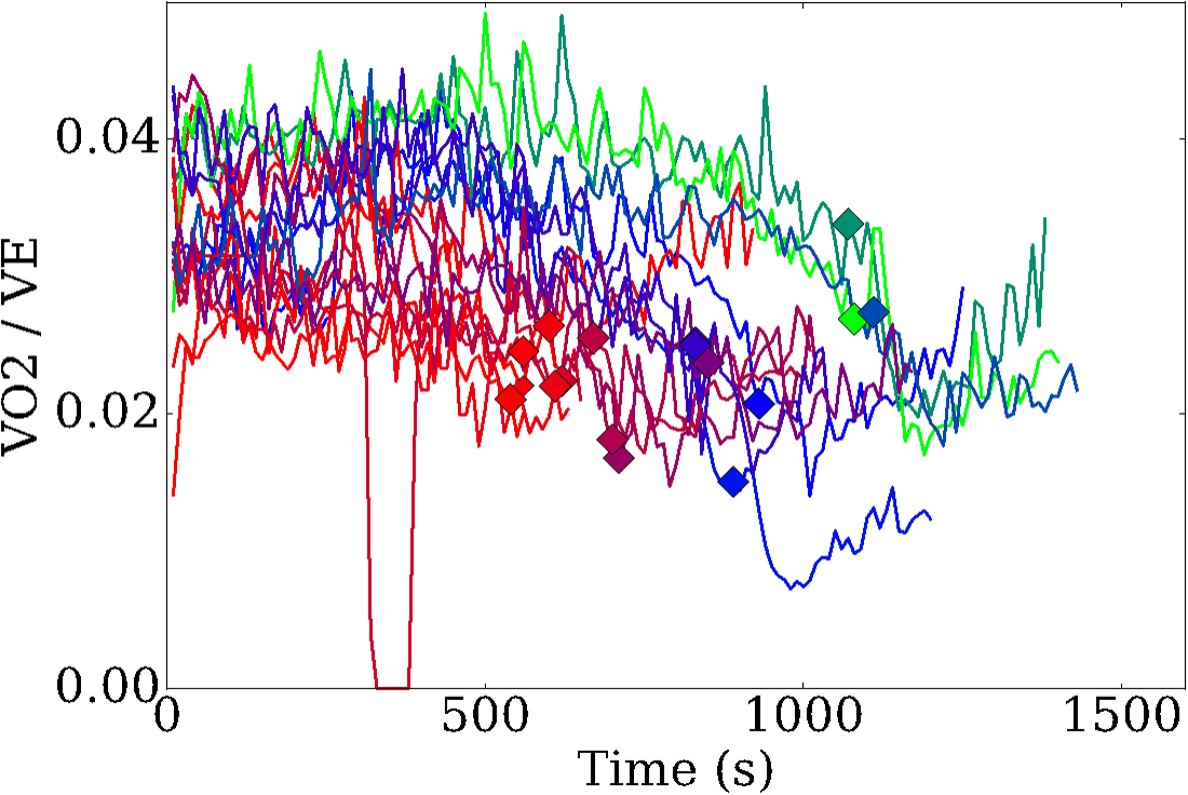
Ratio of oxygen utilisation and total breathing throughout the test. Markers indicate the volitional exhaustion times for each participant.

In Fig 3, we plot the ratio of ${\dot{\text{V}}\text{O}}_{2}$ over total ventilation $\left({\dot{\text{V}}}_{\text{E}}\right)$ with respect to time. The markers on each of the time traces indicate the time of volitional exhaustion for that participant. There are two features that stand out from this figure. Firstly, participants who perform better have higher ${\dot{\text{V}}\text{O}}_{2}{/\dot{\text{V}}}_{\text{E}}$ ratios, suggesting that their oxygen uptake is more efficient than their poorer performing counterparts. Secondly, in the recovery phase of the test (5 minutes following volitional exhaustion) better performing participants exhibit a sharp decrease in ${\dot{\text{V}}\text{O}}_{2}{/\dot{\text{V}}}_{\text{E}}$, which is not observed in the poor performance group. Again, this suggests a more efficient utilisation amongst the former group and that exhalation of CO_2_ is perhaps more significant to total breathing following the test.

**Fig 4:**
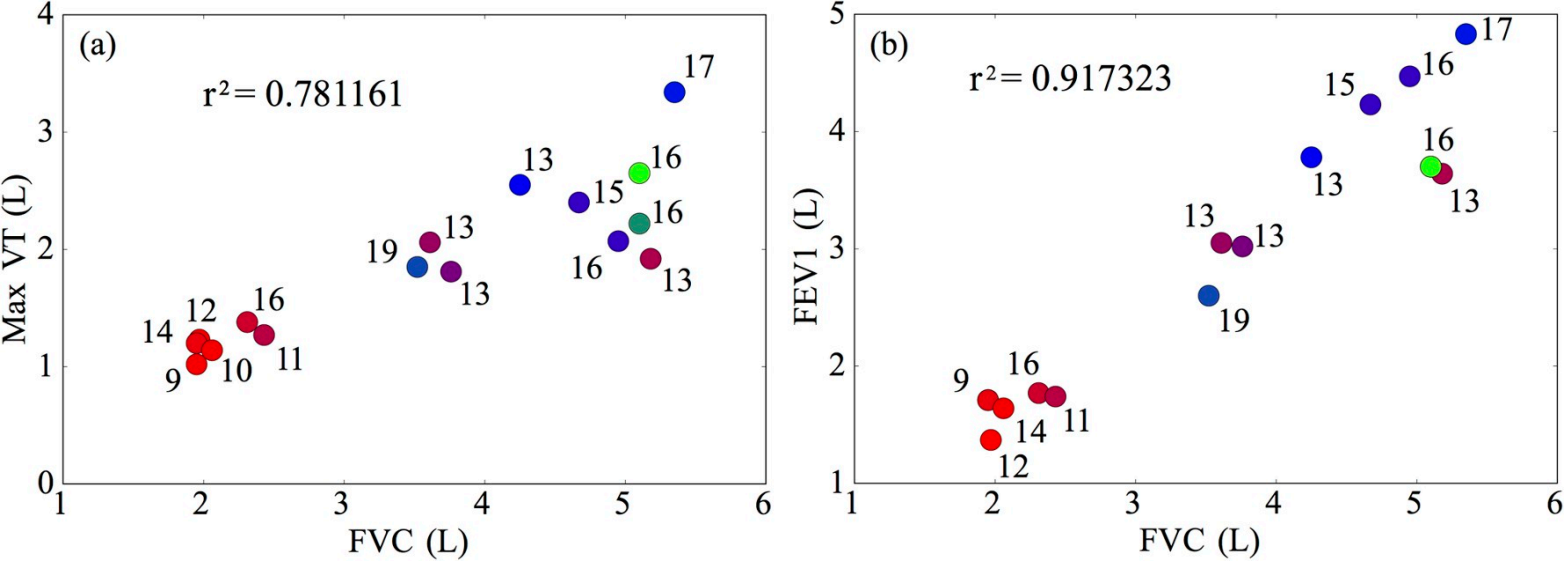
(a) Correlation of FEV_1_ with the maximal tidal volume achieved throughout the test. (b) Correlation between FEV_1_ and FVC is high. Note that, although FVC and FEV_1_ are good predictors of poor test performance, they are unable to distinguish better performing participants.

We next examine the effect that breathing patterns have on participant performance. Two classical prognostic measures used for patients with cystic fibrosis are the forced vital capacity (FVC), and the forced expiratory volume in one second (FEV_1_). These measures have been shown to be well correlated with mortality and overall fitness of CF patient groups [43–45]. In Fig 4, we demonstrate how these metrics are correlated with performance in the CPET test. In Fig 4(a), we observe good correlation between FVC and the maximum tidal volume (VT) of breathing achieved throughout the test. This is unsurprising since participants are likely to be trying to maximise their breathing depth close to their exhaustion point. However, notice that, although the group with low FVC performed poorly, this measure was unable to separate other participants. Fig 4(b) reiterates this result and also highlights the high correlation between FEV_1_ and FVC.

**Fig 5:**
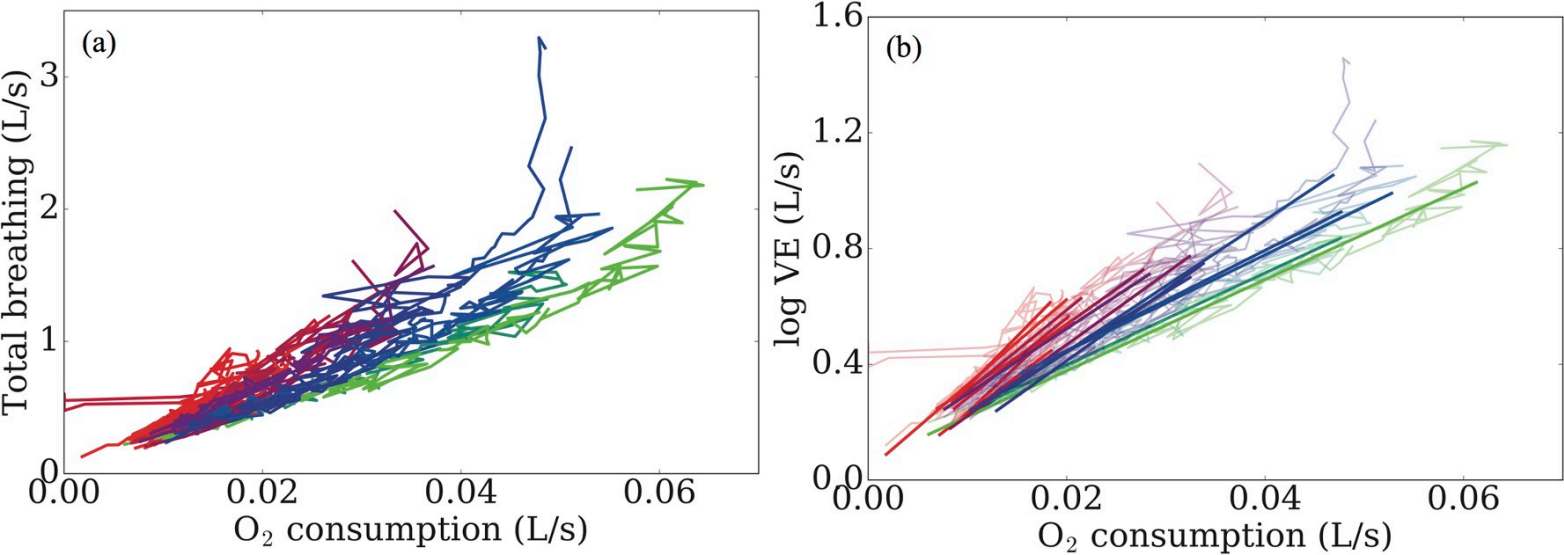
(a) Total ventilation plotted against oxygen utilisation. We observe that breathing pattern is strongly correlated with test performance. (b) Exponential curves are fitted through the raw data, further highlighting this dependence.

In order to better classify the performance of the participants, we must instead look for other factors. In Fig 5, we present the total breathing rate against the oxygen consumption throughout the test. In Fig 5(a), we find a strong relationship between test performance and respiratory pattern. Note that the curvature of the graphs suggests that an exponential fit, rather than a linear one, is most appropriate for these data. In order to test this, we take logarithms of the data and perform a linear regression, ignoring the first 180s of the test since participants are here in the warm up phase (work rate is not increasing) and the final 60s of the data prior to volitional exhaustion, since participants pass their respiratory compensation point, inducing hyperventilation and erratic breathing. The results of the fit are shown in Fig 5(b), where we can clearly see the dependence of performance on breathing pattern.

**Fig 6:**
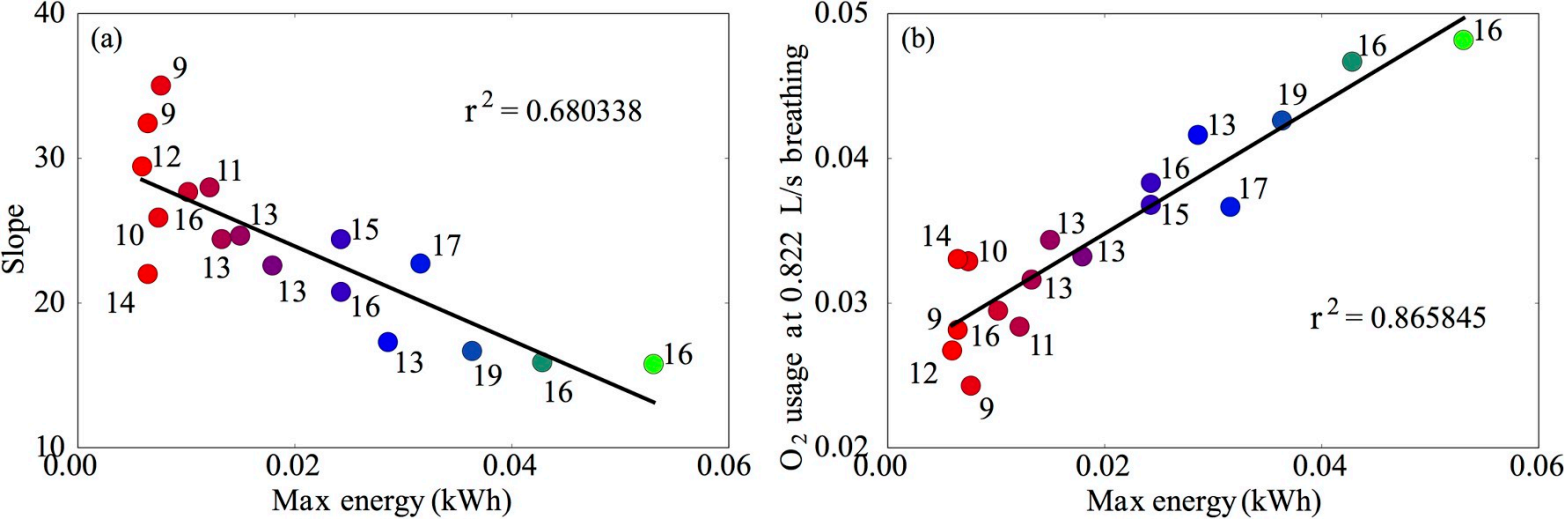
Slope of the fitted curves (${\text{log}\dot{\text{V}}}_{\text{E}}$ against ${\dot{\text{V}}\text{O}}_{2}$) from Fig 4(b) plotted against the total energy transfer during the test. We find a relatively poor characterisation of the variance between performances. (b) By instead plotting the oxygen consumption at a fixed rate of breathing, we better capture differences in performance.

From the fitted curves, we can further explore the dependence of breathing patterns on performance. Firstly, in Fig 6(a), we depict the slope of the fitted curve against the total energy transfer. We find that the slope of the curve of ${\text{log}\dot{\text{V}}}_{\text{E}}$ against ${\dot{\text{V}}\text{O}}_{2}$ alone does not capture all of the variation in energy, which is highlighted by the relatively low coefficient of determination (0.68). Instead, we plot in Fig 6(b) the oxygen consumption at a fixed rate of breathing against the total energy. Here, we find a very good characterisation of the overall performance, with a much higher coefficient of determination (0.86), confirming that those who utilise oxygen more efficiently perform better.

**Fig 7:**
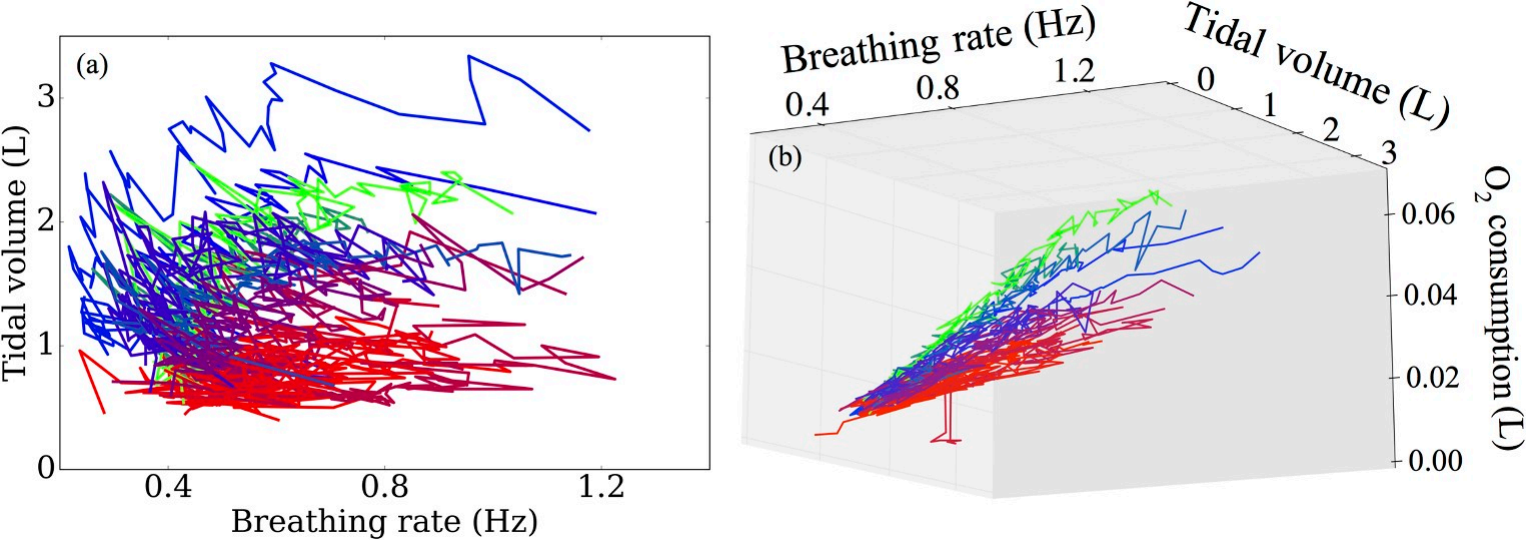
(a) Breathing patterns subdivided into the breathing rate and tidal volume. These data appear uninformative for predicting test performance. (b) With the additional inclusion of the oxygen consumption at a fixed rate of breathing, we find that these variables now almost perfectly capture variation in participant performance.

Next, we examine the specific patterns of breathing exhibited by the participants, in particular, focussing on breathing depth and frequency. Initial characterisations of these patterns seem to provide little information, as indicated in Fig 7(a). However, when we now also include dependence of oxygen consumption, we find a near perfect classification of participants into the lowest performing groups, the best performing groups and the middle group. These data are shown in Fig 7(b). Note that in this figure, the trajectories appear to be evolving on a planar manifold, suggesting significant co-dependence between these three variables.

**Fig 8:**
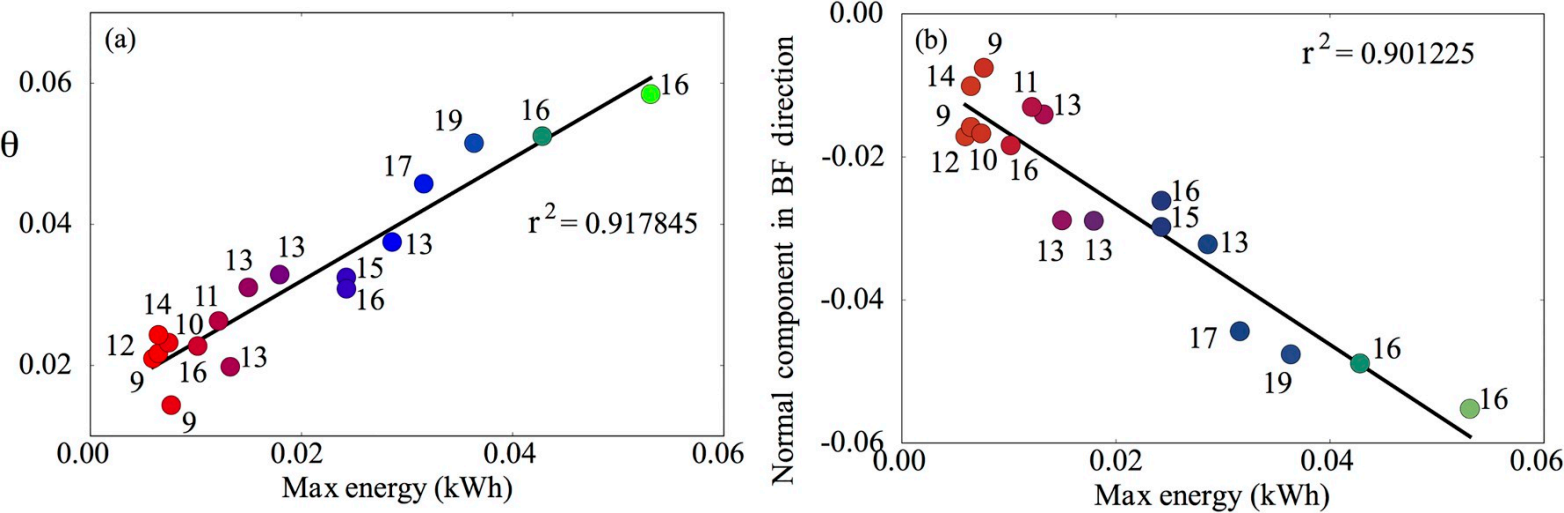
The first principal component obtained via PCA accounts for over 90% of the variation in test performance. (b) Similar levels of variance are accounted for by taking only the normal component of the first principal component, θ, in the breathing frequency direction.

Given that there appears to be co-dependence between the variables used in Fig 7(b), a natural next step is to use principal component analysis (PCA) to account for these dependencies. By projecting the data onto their principal components, we show in Fig 8 how well these components capture the variation in participant performance. Given that there are only three independent variables in our analysis, it is convenient to use spherical polar coordinates to show how these quantify performance. The first of these components, θ, captures over 90% of the variation in performance (Fig 8a), as does the normal component in the direction of breathing frequency (Fig 8b). These results further indicate the importance of breathing frequency, together with co-variation of oxygen consumption and tidal volume as predictors of test performance.

### Gas exchange threshold

Under steady state levels of exercise, the metabolic rate of production of CO_2_ is assumed to be proportional to the utilisation rate of O_2_ via the cellular respiratory quotient, since (after the initial rest-work transition) adenosine triphosphate (ATP) is replenished primarily via aerobic metabolism pathways. As the work rate increases, this pathway becomes unable to supply sufficient ATP to satisfy the required amount of energy and anaerobic pathways have to contribute to overcome the shortfall. In so doing, they increase the levels of metabolic waste products, such as lactate and also increase the overall production rate in CO_2_. The point at which this occurs is known as the anaerobic threshold (AT) or sometimes can be referred to as the lactate threshold. The lactate threshold can be estimated non-invasively by the determination of the GET [46]. Within this study, the GET was successfully identified in all CPETs.

**Fig 9:**
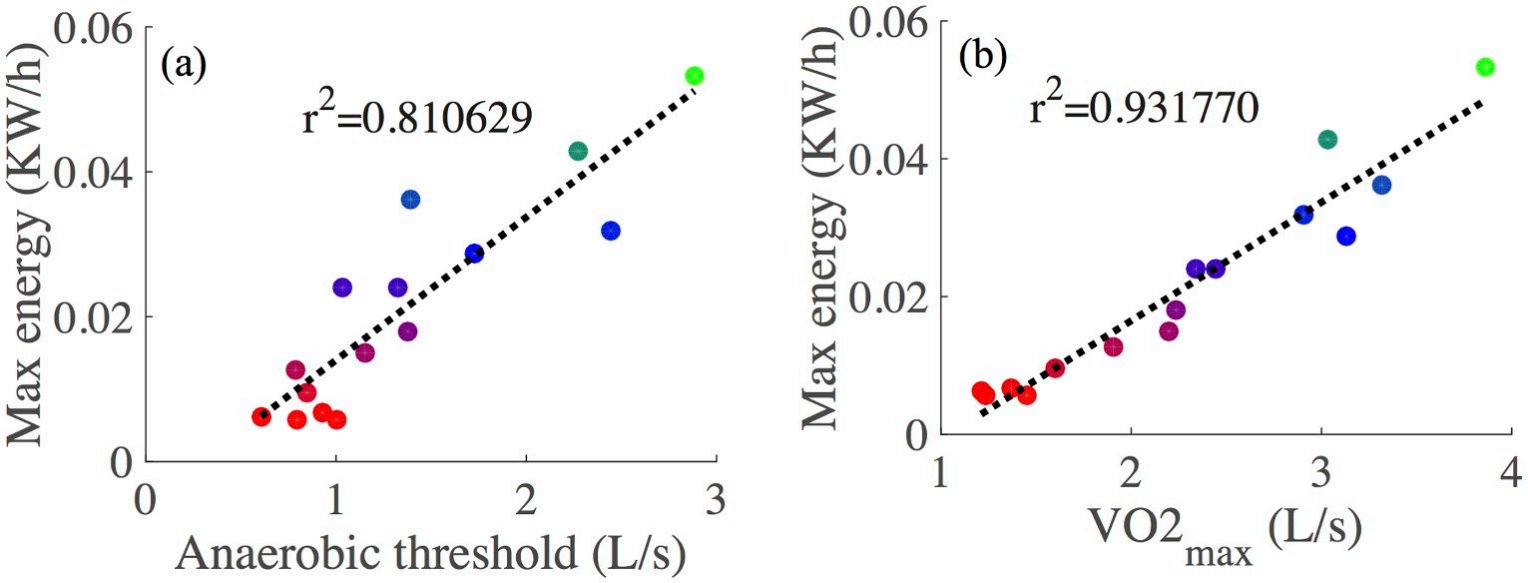
(a) Quantifying the relationship between the anaerobic threshold and overall test performance. Thresholds were calculated using an automated procedure based on previous methods [46] (b) ${\dot{\text{V}}\text{O}}_{2\text{max}}$ is the best single predictor of overall test performance.

Anaerobic thresholds are well correlated with overall performance, as shown in Fig 9(a), due to the fact that the anaerobic pathways are less efficient at producing ATP and because build-up of lactate contributes significantly to fatigue. One of the major contributing factors in defining GET is ${\dot{\text{V}}\text{O}}_{2\text{max}}$, since this is indicative of the limit of the rate of oxidative phosphorylation. It thus comes as little surprise that ${\dot{\text{V}}\text{O}}_{2\text{max}}$ is the best single predictor of CPET performance, as shown in Fig 9(b).

### Gaussian processes-based modelling

Having completed a detailed data analysis and identified candidate predictors of CPET performance we next use these predictors to inform a Gaussian Process-based model. In our modelling, we attempt to describe the influence of breathing patterns and ${\dot{\text{V}}\text{O}}_{2}$ on the GET, since this is shown to correlate well with overall test performance (see Fig 9). In mathematical terms, we treat GET as our scalar output variable, with input variables comprising: baseline breathing rate and tidal volumes, and O_2_ consumption rates at a fixed ventilation rate, FVC, FEV_1_, and the rate of changes in breathing rate and tidal at exercise onset, using the slopes calculated based on the analysis presented in Fig 5(b). Thus, we have an output variable that dependents on seven input variables, which we store in a vector **x $\in D\subset R^{d}$**, with *d =* 7. We now assume that there exists a ‘true’ function *f*: *D →R,* such that GET = *f(***x**).

### GP emulator performance

**Fig 10:**
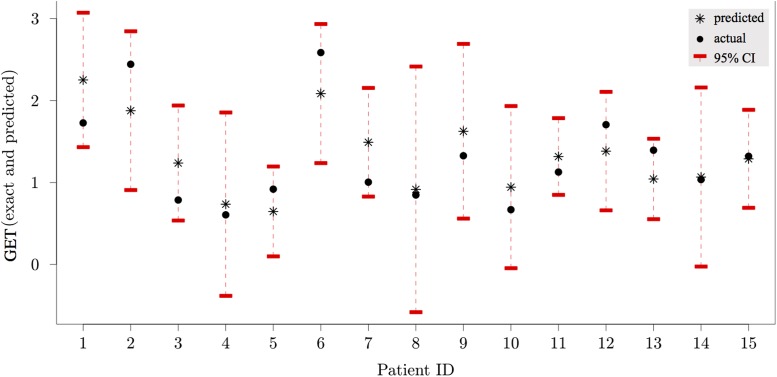
Predictions (asterisks) vs. exact values (bullets). Red bars show the 95% confidence intervals, *m*_i_(x_i_) ± 1.96 *s*_*i*_(x_i_) around the predicted value, where *m*_i_ and *s*_i_ are GP prediction mean and standard deviation based on all but the *i*’th data point.

The GP emulator was constructed using the data presented above, with GET calculated using previously described methods [46]. These data were used to train the emulator. We consider a first order polynomial regression for the mean *μ*(.) and the squared-exponential covariance function for K(.,.). Since in this pilot study, we have a small number of participants, we use leave-one-out cross-validation mean squared error (MSE_LOO_) to assess the accuracy of our emulator. It is defined as

$${MSE}_{LOO}\;=\;\frac{1}{n}\sum_{i\;=\;1}^{n}{\left(m_{-i}(x^{i})-f(x^{i})\right)}^{2},$$

where *m*_−*i*_(***x***^*i*^) is the prediction obtained by the GP model based on all the training data points except the *i’th* one [47]. The corresponding prediction uncertainty is denoted by $s_{-1}^{2}(x^{i})$. In this work, *MSE*_*LOO*_ = 0.1676 which is around 12% of the global mean for GET. Fig 10 shows exact values (bullets) vs. *m*_−*i*_(***x***^*i*^) (asterisks) with the 95% confidence intervals, i.e. $m_{-i}(x^{i})\pm 1.96s_{-i}(x^{i})$; for each patient, we use the emulator trained against the remainder of the training data to approximate the AT value for that patient, given their input variables.

The emulator has reasonable accuracy, in spite of the small number of data used to train it. In general, for high accuracy in GP emulator, the number of sample training data (corresponding to the number of patients in our case) should be around ten times larger than the number of input variables [48]. Clearly, there is a need to acquire further data points to improve the predictive capabilities of the emulator. For each patient, the 95% confidence interval around the predicted point contains the true value. However, the large variance in the GP estimates for some patients (e.g. patient 8) highlights a need to extend this study to include more data to refine estimates around these points, particularly to deal with the high variability of lung function parameters in this patient group [49].

### Combining patients’ data with mechanistic modelling

An alternative to the Gaussian Process-based modelling approach above would be to directly use the experimental data in a mechanistic modelling framework. Next we present an example of employing this alternative approach. During exercise, it is known that dead-space ventilation rate, ${\dot{\text{V}}}_{\text{D}}$ increases [50]. Thin et al [50], demonstrated that this increase is significantly greater in CF patients. Physiologically this is because, in CF patients, tidal volume, V_T_ is limited. Therefore, there must be an increase in the frequency of breathing, BF, in order to maintain gas exchange.

**Fig 11:**
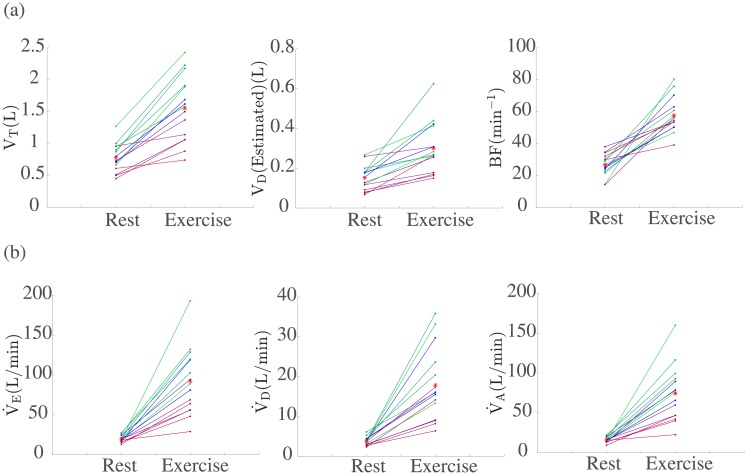
(a) Tidal volume (V_T_), an estimate for the dead volume (V_D_) and Breathing frequency BF from the data. (b) Changes in ventilatory parameters during exercise for the 15 CF patients using the proposed form of ${\dot{\text{V}}}_{\text{D}}$ = ${\dot{\text{V}}}_{\text{DB}}$ + *a* × W(t). In the case of ${\dot{\text{V}}}_{\text{E}}$ the solid dots represent values extracted from the data, for ${\dot{\text{V}}}_{\text{D}}$ and ${\dot{\text{V}}}_{\text{A}}$ these represent values taken from model simulations (see S1 File). In all cases the red stars represent the group mean.

Fig 11(a) shows the changes in these ventilatory parameters at rest and during peak exercise for the 15 CF patients. This figure indicates that tidal volume is decreased in the CF patients as the disease severity increases. Thus, it seems reasonable to suggest that the size of increase in ${\dot{\text{V}}}_{\text{D}}$ with exercise is dependent on the severity of the disease. We can now consider this in the case of the 15 CF patients tested. For each of these patients a work rate was prescribed dependent on their previous performances (see Fig 2). We incorporate the work rate data in the mechanistic model by Timischl [51] in order to predict ${\dot{\text{V}}}_{\text{E}}$ for each individual patient (see S1 File for details on the mechanistic model). It is important to note that Timischl’s original model ignores the dead-space (i.e. assumes ${\dot{\text{V}}}_{\text{D}}$ = 0). Figure A in S1 File shows that generally for CF patients whose disease is less severe (those in green) ${\dot{\text{V}}}_{\text{E}}$ is captured better with no ventilatory dead-space than those whose disease is more severe. We therefore suggest incorporating the dead space by modelling ${\dot{\text{V}}}_{\text{D}}$ using a linear function of the form, ${\dot{\text{V}}}_{\text{D}}$ = ${\dot{\text{V}}}_{{\text{D}}_{\text{B}}}$ + *a* × W(t), where ${\dot{\text{V}}}_{\text{DB}}$ is the base level of ${\dot{\text{V}}}_{\text{D}}$ for each patient, *a* is a scaling parameter based on the severity of the disease and W(t) is the work rate. We anticipate that for those patients whose disease progression is less severe a relatively low scaling factor will be needed and for those whose condition is more severe, a much larger one. To determine the values of ${\dot{\text{V}}}_{\text{DB}}$ and *a* we use the available experimental data. It is known that, ${\dot{\text{V}}}_{\text{D}}$ = BF × V_D_. As shown in Fig 11(a) the available data allows us to obtain values for BF and V_D_ both at rest and during exercise. We can therefore determine a value for ${\dot{\text{V}}}_{\text{DB}}$ as follows, ${\dot{\text{V}}}_{\text{DB}}$ = BF (Rest) × V_D_ (Rest), and a value for *a* as follows, *a* = (BF (Exercise) × V_D_ (Exercise)) / W (Exercise). Next, we simulate the model with this added ventilatory dead-space (model predictions are shown in Figure B in S1 File). This allows us to obtain predictions for the total ventilation (${\dot{\text{V}}}_{\text{E}}$) for each individual patient as shown in Figure B in S1 File. The results from these simulations in turn allow us to predict alveolar ventilation, ${\dot{\text{V}}}_{\text{A}}$, for each patient as shown in Fig 11(b). In Fig 11(b) we plot ${\dot{\text{V}}}_{\text{E}}$ from the data, along with the predicted by the modified model simulations values for ${\dot{\text{V}}}_{\text{D}}$. Then using ${\dot{\text{V}}}_{\text{A}}$ = ${\dot{\text{V}}}_{\text{E}}$ − ${\dot{\text{V}}}_{\text{D}}$ to find ${\dot{\text{V}}}_{\text{A}}$, we can predict the changes in ${\dot{\text{V}}}_{\text{A}}$ during exercise for the 15 CF patients.

This is an example of how individual patient’s data could be used in a mechanistic model framework in order to predict individual patients’ characteristics, in this case associated with ventilatory parameters and hence lung function.

## Discussion

Our primary long-term modelling aim is to eventually use the models to evaluate how CF impairs exercise tolerance relative to increasing ventilatory and metabolic demands. Our predictive models could also be used to evaluate therapies and their effect on exercise performance. Ultimately, we hope that this will form a series of steps to design better exercise treatment that is tailored to specific individual needs relative to patient’s treatment therapies, a treatment modality that is affordable, and personalised [52]. It is interesting to note that in spite of the small number of data points used to train our GP emulator, the accuracy as computed by the leave-one-out cross-validation mean square error is high. This observation implies that the relationship between GET and our chosen input variables is ‘smooth’ (in that there are no large, sudden changes in GET as our input variables vary) [53]. In turn, this provides further evidence that our chosen input variables are good predictors of GET for this patient group.

The data analysis and modelling results have highlighted the dependence of CPET performance on variables associated with ventilation and metabolic rates of O_2_ consumption. We believe that this is the first attempt to mathematically model the relationship between ventilatory dynamics, ${\dot{\text{V}}\text{O}}_{2}$ and performance in CPET within a group of children and adolescents with CF. Whilst it is clear that there is much work to be done in this area, we hope that this will serve as a starting point for improved modelling for CF, not only in the arena of GP emulators, but also in the domain of mechanistic modelling, which we shall describe briefly. But one of the benefits of modelling includes the ability to utilise existing data sets at a time when there are limited resources and time for a patient group who can be very sick and unable to engage in research.

### Perspectives for GP improvements

In our simulator, we have used the GET as our output (dependent) variable. Another choice for this could be the performance in the CPET or ${\dot{\text{V}}\text{O}}_{2\text{max}}$_,_ since these are the primary biomarkers for gauging aerobic fitness. However, the use of the GET has been shown to have high agreement with the lactate threshold (another surrogate for the AT), and related to disease severity in CF [54]. Furthermore, as reported in Fig 9(a), the GET location for a given patient correlates well with their overall performance in this test. Importantly, by constructing a predictive model to approximate the GET values for a patient, we can hope to further extend this to identify contributions of aerobic and anaerobic pathways in supplying ATP to meet the demand imposed during the exercise test.

The initial exploration of results highlighted that both ventilation parameters and metabolic rates of O_2_ consumption were the primary factors influencing test performance. It is clear that ${\dot{\text{V}}\text{O}}_{2}$ should play a significant role in determining the GET location, since it is a proxy for oxidative phosphorylation which is the main pathway for ATP synthesis in steady state exercise. As a measure of oxygen uptake efficiency in our model, we use the oxygen consumption rate at a fixed total ventilation rate (that being 0.822 L·s^-1^) as an input (independent) variable for each patient.

There are a number of ventilatory input variables incorporated in our simulator. Given their potential importance as clinical biomarkers, highlighting the limitations of lung capacity and function, we include FVC and FEV_1_ as input (independent) variables. During the aerobic exercise test, participants spend three minutes cycling at a minimal work rate, over which we quantify their baseline breathing frequency and baseline tidal volume by taking the means of these variables over this period. To capture the dynamics response associated with the exercise, the rates of change of breathing frequency and tidal volume are calculated, based on the fits obtained in Fig 5(b). The rates of change of these ventilatory variables indicate how individual participants respond to changes in work rate and were shown in Fig 7(b) to discriminate between participant performances. Moreover, differences in rates of change of breathing frequency and tidal volume have previously been shown to be significantly different between control groups and CF groups [50], suggesting that these are potentially key biomarkers for assessing aerobic fitness in patients with CF.

At present, the GP model is conditioned on specific data points for each patient. An improvement to the GP could be made by instead conditioning with respect to distributions. Given that repeated tests are often performed for the same individual, so that multiple sample points are provided for each participant, we can consider a fit to a probability distribution capturing the variability in the identified variables. This approach has advantages compared to standard GP models, such as avoiding problems associated with over fitting and regularisation (which is important for the inverting ill-conditioned covariance matrices that often arise during the application of [1, 2, 55]).

### Perspectives for mechanistic modelling

In order to better understand and characterise the difference between performances, it would be extremely informative to construct and simulate a mechanistic mathematical model, based upon on an ordinary differential equation (ODE) framework, describing the relationship between the cardiopulmonary system and the metabolic dynamics of skeletal muscle. By describing the relationships between different organ-level systems, the model would be able to identify the patient-specific rate-limiting factors defining aerobic fitness. Moreover, analysis of the model could be used to suggest treatment strategies to improve these factors and thus predict how patients will improve under such regimes.

At the individual organ level, there are a plethora of models describing individual dynamics of the level of the heart [56–59], lung [60–63] and systemic metabolic demand [64–70]. There also exist a number of models describing such interactions between cardiopulmonary and metabolic systems [51, 71–74] in a variety of settings, including heart failure and mechanical ventilation. A core feature in all of these models is the nonlinear interactions between the constituent model compartments that encompass the distinct tissues. An important consequence of this is that the model must be studied as whole, in an integrated fashion, to truly understand the body’s response to exercise.

With respect to the present question, there are a number of limitations of the existing modelling approaches. Most significantly, none have been designed with either an adolescent, or a CF patient group in mind, and the nuances of these patient groups will have to be factored into to any model development. In particular, these models have relatively simple, empirical models to describe changes in ventilation, which may not capture well the breathing dynamics of our patient group. Moreover, to the best of our knowledge, no model considers the changes in ventilation separated into breathing frequency and depth that have been shown by us and others [50] to be critical to overall test performance.

In our analysis, we have demonstrated that the GET location is a critical factor in determining overall patient aerobic fitness. Many of the mathematical exercise models describe only steady-state exercise, in which aerobic pathways meet most of the ATP demand [51, 71–74]. As such, these models are inadequate to capture the dynamics we describe here. Another common topic of study is the dynamic response at exercise onset, which again, does not meet the current need to describe the GET crossing point [75–78].

Of the mathematical models that describe the contribution of anaerobic pathways to ATP production, some assume that the shortfall in meeting ATP demand via oxidative phosphorylation is met entirely by anaerobic pathways [79], yet this is clearly not so, since ATP levels in skeletal muscle post-exercise may be up to 30% lower than pre-exercise values [80]. Mathematical models that factor in fatigue brought about by anaerobic metabolism are generally phenomenological in nature, and it is difficult to quantify these models against real patient data [79, 81, 82]. Moreover, these models mostly fall outside the arena of ODE-based modelling and so dynamical properties are difficult to infer from them.

**Fig 12:**
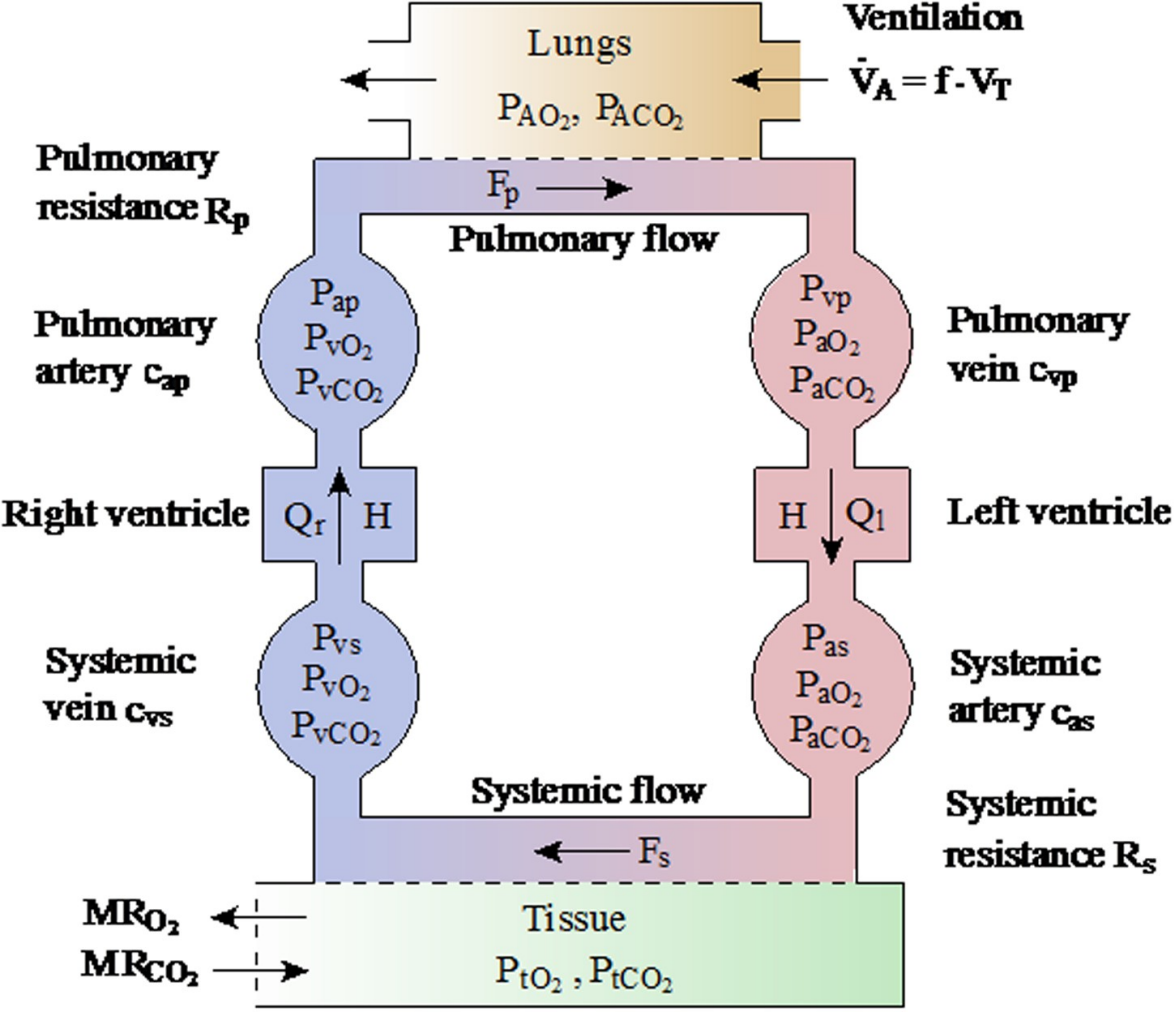
Schematic of the variables and processes in the proposed ODE-based mathematical model. Adapted from Timischl [51] and Batzel *et al*. [86].

Developing a mathematical model to describe the integrated behaviour of all of the relevant organs, whilst remaining biophysically plausible, but without requiring excessive or invasive parameterisation, is a difficult task. The proposed model should include descriptions of the cardiovascular system, the ventilatory system and simple models of metabolism at the tissue level. Specifically, dynamic variables should include alveolar, arterial, venous and tissue level partial pressures/concentrations of O_2_ and CO_2_, cardiac output, ventilation and metabolic rates oxygen utilisation and CO_2_ production. Partial alveolar gas pressures can be linked to data collected during the test, and the work rate can then be provided as inputs to the model. Note that these variables are similar to those included in previously defined models [51, 71–74] and the aim is to extend these to describe the dynamics observed in patients with CF. The proposed model schematic is displayed in Fig 12.

Of critical importance to the overall model construction is the development of a simple, yet realistic model of cellular metabolism, to overcome the issues discussed earlier. The model should respect the different metabolic processes that occur in the muscle tissue, in particular: glycolysis, phosphocreatine breakdown and synthesis and oxidative phosphorylation, in a simplistic fashion that is amenable to being fit to CPET data. Whilst there are models that describe the biochemical reactions associated with these processes, and importantly, their stoichiometry [65, 68, 70, 73, 83], quantifying their associated rate constants *in vivo* is a near-impossible task, and so efforts must be made to develop a model that incorporates the relevant metabolic dynamics whilst being simple enough to be fit to data.

With knowledge of the integrated system, attempts can also be made to describe other important exercise-based processes, such as lactate buffering and recycling (as a fuel source) [68, 84, 85] and the overall muscle fatigue brought about by the combination of all of these factors. Only by systematically exploring the dependence of aerobic fitness of all of the factors described in this section can we begin to understand the system in an integrated fashion.

## Limitations

A limitation with the current study is the utilisation of a relatively small sample size, and this is most likely contributing towards aforementioned errors in prediction. Future studies should seek to utilise CPET collected annually in CF centres, to develop larger, multi-centre, samples whereby a uniform exercise protocol is utilised. Given that utilisation of CPET is now recommended and endorsed for regular use by international medical societies [87], and individual CF centres are reporting upon experiences of using CPET [88], large-scale utilisation of such data is a feasible target.

The findings presented here are derived from a smaller sample, and therefore the models presented are only preliminary models of this patient cohort; however, our study provides a unique examination into the aerobic and anaerobic signatures of individual patients with CF in response to progressive exercise.

As models improve, as well as the quality of fits to data (through increased sample sizes), these can be used in a prognostic setting to predict potential improvements in aerobic fitness that may arise due to therapeutic intervention. Moreover, with proper mechanistic modelling of the primary organs affected in CF, there exists the potential to optimise treatment for this patient group by identifying the limiting factors of aerobic fitness. Finally, whilst this study provides an insight into metabolic process during exercise, future research and models must account for additional variables predictive of function and mortality (e.g. genotype, body composition, pancreatic sufficiency, infection status, exacerbations [89, 90]) and co-morbidities (CF-related diabetes [91], pulmonary arterial hypertension [92]) existent within CF, notably those that may affect exercise tolerance.

## Conclusions

In conclusion, our proposed stimulator allows for the reproduction of the physiological observations of ventilation and metabolic rates of O_2_ consumption acquired during a CPET in relation to people with CF. The modelling framework was able to successfully replicate the relationship between ventilatory dynamics, lung capacity and function and performance in CPET within a group of children and adolescents with CF. In particular, by using the Gaussian processes (GP) the GET, a well-known physiological threshold marker of exercise intensity, as well as other important measurements such as breathing frequency and tidal volume, at the individual patient level were accommodated into the model. The stimulator has the potential to be suitable for future applications of the investigations of drug therapies or other physical interventions on exercise performance.

## Supporting information

S1 FileCardiopulmonary responses to maximal aerobic exercise in patients with cystic fibrosis.**Figure A**: Model simulations of ${\dot{\text{V}}}_{\text{E}}$ assuming V_D_ = 0, compared to the recorded data value of ${\dot{\text{V}}}_{\text{E}}$ obtained during the CPET. The thin red line gives ${\dot{\text{V}}}_{\text{E}}$ as measured from the data and the bold coloured line gives ${\dot{\text{V}}}_{\text{E}}$ as simulated by the model. The colour given to each patient is the same as given in Fig 2 in the main text of the paper. **Figure B**: Model simulations of ${\dot{\text{V}}}_{\text{E}}$ using the proposed linear model of V_D_, compared to the recorded data value of ${\dot{\text{V}}}_{\text{E}}$ obtained during the CPET. The thin red line gives ${\dot{\text{V}}}_{\text{E}}$ as measured from the data and the bold coloured line gives ${\dot{\text{V}}}_{\text{E}}$ as simulated by the model. The colour given to each patient is the same as given in in Fig 2 in the main text of the paper. Fitted parameter values can be found below. **Table A**: Parameter values. **Table B**: Calculated values of ${\dot{\text{V}}}_{{\text{D}}_{\text{B}}}$ and *a* for the linear model of ventilatory dead-space. **Table C**: Definitions of model parameters.(PDF)
