# Supplementary material for: Cardiopulmonary responses to maximal aerobic exercise in patients with cystic fibrosis
Source: PLoS One. 2019 Feb 13;14(2):e0211219. doi: 10.1371/journal.pone.0211219 (PMC6373911; doi:10.1371/journal.pone.0211219)
Supplement: S1 File — Figure A: Model simulations of V˙E assuming VD = 0, compared to the recorded data value of V˙E obtained during the CPET. The thin red line gives V˙E as measured from the data and the bold coloured line gives V˙E as simulated by the model. The colour given to each patient is the same as given in Fig 2 in the main text of the paper. Figure B: Model simulations of V˙E using the proposed linear model of VD, compared to the recorded data value of V˙E obtained during the CPET. The thin red line gives V˙E as measured from the data and the bold coloured line gives V˙E as simulated by the model. The colour given to each patient is the same as given in in Fig 2 in the main text of the paper. Fitted parameter values can be found below. Table A: Parameter values. Table B: Calculated values of V˙DB and a for the linear model of ventilatory dead-space. Table C: Definitions of model parameters. (PDF) [file pone.0211219.s001.pdf]

Supplementary Information for CARDIOPULMONARY RESPONSES TO MAXIMAL AEROBIC EXERCISE IN PATIENTS WITH CYSTIC FIBROSIS

Craig A. Williams* Kyle C. A. Wedgwood† Hossein Mohammad‡ Katie Prouse§
Owen W. Tomlinson¶ Krasimira Tsaneva-Atanasova‖

January 30, 2019

An empirical relation for ventilation

A global model of the human cardio-vasular and respiratory system is developed in [1]. Given a prescribed workload we may use this to predict how an individual will physiologically respond. Although many physiological factors are of importance when determining an individual's fitness and prognosis, gas exchange during exercise is of particular interest.

The total ventilation $\dot{V}_E$ can be divided into alveolar ventilation $\dot{V}_A$ and dead-space ventilation $\dot{V}_D$.

$$\dot{V}_E = \dot{V}_A + \dot{V}_D$$

In every breath, a certain proportion of the air does not take part in gas exchange; this is the dead-space ventilation. For patients with cystic fibrosis, during exercise a large proportion of total ventilation is wasted.
Total ventilation, as in Timischl's model [1], can be described by the empirical relation

$$\dot{V}_E = G_p e^{-0.05 P_{a_{O_2}}} (P_{a_{CO_2}} - I_p) + G_c (P_{a_{CO_2}} - I_c)$$

*Children's Health and Exercise Research Centre, Sport and Health Sciences, University of Exeter, Exeter, UK.
†Department of Mathematics and Living Systems Institute, University of Exeter, Exeter, UK and Centre for Biomedical Modelling and Analysis, University of Exeter, Exeter, EX4 4QJ
‡EPSRC Centre for Predictive Modelling in Healthcare, University of Exeter, Exeter, EX4 4QJ, UK
§Department of Mathematics, University of Exeter, Exeter, EX4 4QF, UK
¶Children's Health and Exercise Research Centre, Sport and Health Sciences, University of Exeter, Exeter, UK.
‖Department of Mathematics and Living Systems Institute, University of Exeter, Exeter, EX4 4QF, UK and EPSRC Centre for Predictive Modelling in Healthcare, University of Exeter, Exeter, EX4 4QJ, UK

A model with no dead-space

If we start by assuming that dead-space ventilation is so small that we may neglect it's contribution to $\dot{V}_E$, thus assuming $\dot{V}_E = \dot{V}_A$. Then using the empirical form for $\dot{V}_E$, we could use Timischl's model to give predictions of how each individual will respond to their prescribed workload.

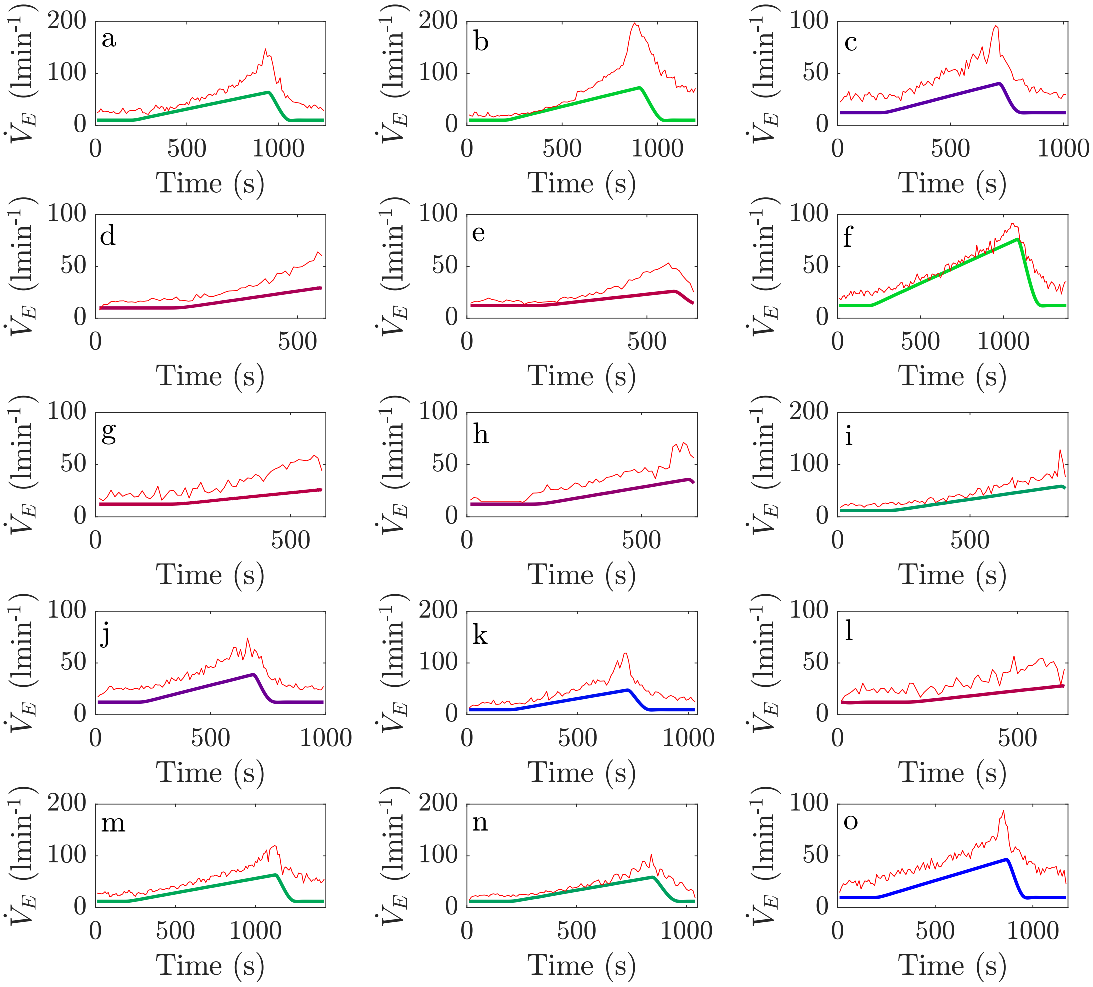

Fig. A: Model simulations of $\dot{V}_E$ assuming $\dot{V}_D = 0$, compared to the recorded data value of $\dot{V}_E$ obtained during the CPET. The thin red line gives $\dot{V}_E$ as measured from the data and the bold coloured line gives $\dot{V}_E$ as simulated by the model. The colour given to each patient is the same as given in **Fig. 2** in the main text of the paper.

From the model simulations in **Fig. A** we can see that for many of the patients assuming $\dot{V}_E = \dot{V}_A$ gives a reasonably good fit to the data. In all cases the shape is generally similar, but in some cases the scaling of the graphs does seem to differ.

A linear model for ventilatory dead-space

During exercise, it is known that dead-space ventilation, $\dot{V}_D$ increases [2]. Thin et al. [2], demonstrated that this increase is significantly greater in the CF patient. Physiologically this is because, in the CF patient tidal volume, V_T, is limited. Therefore, there must be an increase in the frequency of respiration, BF, in order to maintain gas exchange, this is what leads to the increase in $\dot{V}_D$ in the CF patient.

Now, it is observed that tidal volume is decreased in the CF patient as the disease severity increases. Thus, it seems reasonable to suggest that the size of increase in $\dot{V}_D$ with exercise is dependent on the severity of the disease. We can consider this in the case of the 15 CF patients tested.

For each of these patients a work load was prescribed dependent on their previous performances. Therefore, those individuals who have performed well in previous tests, and therefore are presumably 'fitter'. Patients in green were those who performed best in previous tests and it is therefore reasonable to assume that their progression of the disease is less than those in red. Looking at **Fig. A**, we can see that generally, the CF patients whose disease is less severe (those in green) is modelled better with no ventilatory dead-space than those whose disease is more severe.

We therefore suggest modelling $\dot{V}_D$ by a function of the form;

$$\dot{V}_D = \dot{V}_{D_B} + aW(t)$$

where $\dot{V}_{D_B}$ is the base level $\dot{V}_D$ for each patient and a is a scaling parameter based on the severity of the disease in the patient. For those patients whose disease progression is less severe the relatively low scaling factor will be needed and for those whose condition is more severe, a much larger one.

To determine the values of $\dot{V}_{D_B}$ and a we may make use of the data.

$$\dot{V}_D = \mathrm{BF} \times V_D$$

Our data gives values of BF and V_D both at rest and during exercise (see **Fig. 11** in the main text). We can therefore determine a value for $\dot{V}_{D_B}$:

$$\dot{V}_{D_B} = \mathrm{BF}_{\mathrm{Rest}} \times V_{D_{\mathrm{Rest}}}$$

and a value for a:

$$a = \frac{\mathrm{BF}_{\mathrm{Exercise}} \times V_{D_{\mathrm{Exercise}}}}{W_{\mathrm{Exercise}}}$$

Now running the model with this added ventilatory dead-space, we can obtain predictions for $\dot{V}_E$, as before. These are shown in **Fig. B**.

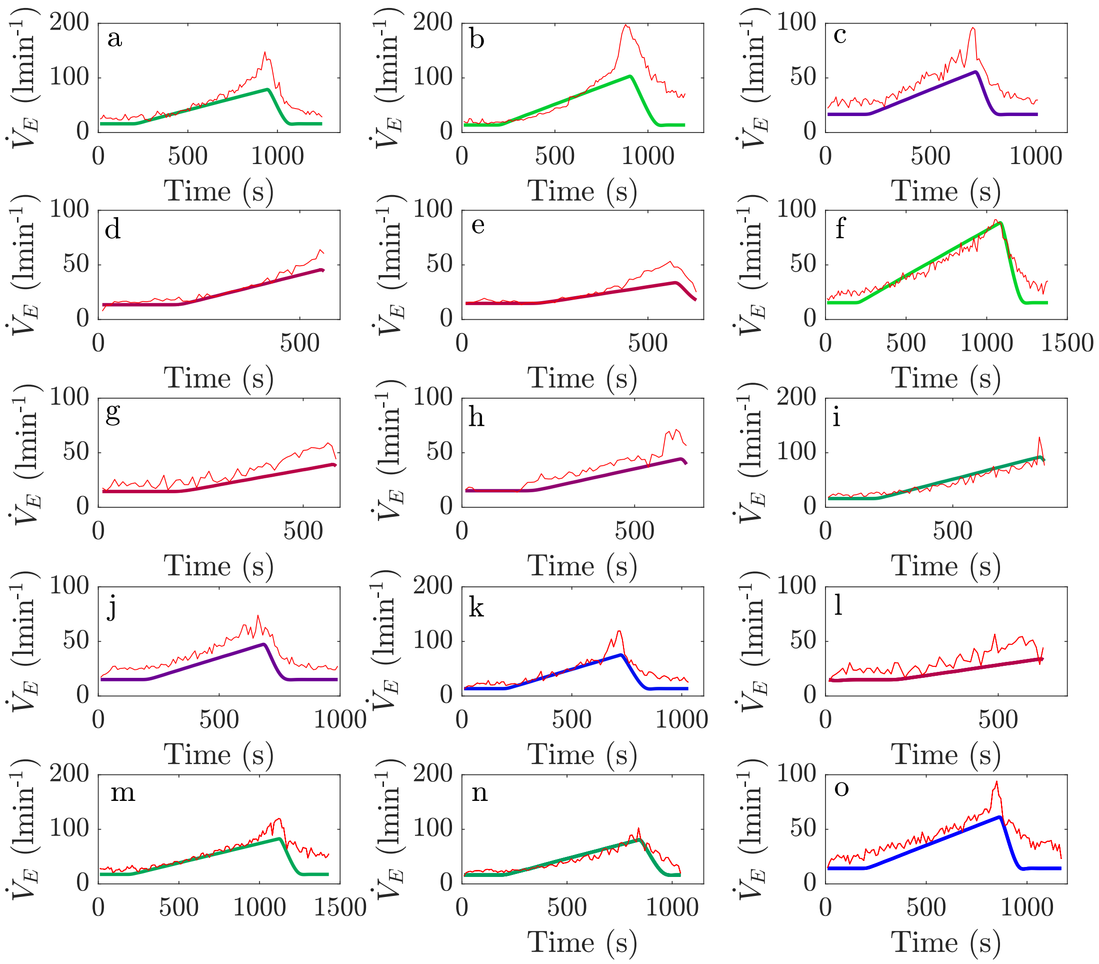

Fig. B: Model simulations of $\dot{V}_E$ using the proposed linear model of $\dot{V}_D$, compared to the recorded data value of $\dot{V}_E$ obtained during the CPET. The thin red line gives $\dot{V}_E$ as measured from the data and the bold coloured line gives $\dot{V}_E$ as simulated by the model. The colour given to each patient is the same as given in in **Fig. 2** in the main text of the paper. Fitted parameter values can be found below.

The results of these simulations are summarised in **Fig. 11** in the main text. The results in this figure are comparable to what has been obtained by Thin et al. [2]. The changes observed within our 15 patient sample are much more extreme than those seen by Thin et al. [2]. This is not a surprise as the patients in our sample were being tested to much higher intensities and as such there is more dependence of BF and thus the more extreme responses to exercise.

Now using this improved form of $\dot{V}_E$ we may run our model once more to gain a better understanding of how other physiological factors change during exercise.

The Timischl's Model

For completeness we include the full model equations here.
The full model, as given by [1] and used in all model simulation and analysis is given.

$$\begin{aligned}
V_{A_{CO_2}}\dot{P}_{a_{CO_2}}(t) &= 863F_p(t)(C_{v_{CO_2}}(t) - C_{a_{CO_2}}(t)) + \dot{V}_A(P_{I_{CO_2}} - P_{a_{CO_2}}(t)),\\
V_{A_{O_2}}\dot{P}_{a_{O_2}}(t) &= 863F_p(t)(C_{v_{O_2}}(t) - C_{a_{O_2}}(t)) + \dot{V}_A(P_{I_{O_2}} - P_{a_{O_2}}(t)),\\
V_{T_{CO_2}}\dot{C}_{v_{CO_2}}(t) &= MR_{CO_2} + F_s(t)(C_{a_{CO_2}}(t) - C_{v_{CO_2}}(t)),\\
V_{T_{O_2}}\dot{C}_{v_{O_2}}(t) &= -MR_{O_2} + F_s(t)(C_{a_{O_2}}(t) - C_{v_{O_2}}(t)),\\
c_{as}\dot{P}_{as}(t) &= Q_l(t) - F_s(t),\\
c_{vs}\dot{P}_{vs}(t) &= F_s(t) - Q_r(t),\\
c_{vp}\dot{P}_{vp}(t) &= F_p(t) - Q_l(t),\\
\dot{S}_l(t) &= \sigma_l(t),\\
\dot{S}_r(t) &= \sigma_r(t),\\
\dot{\sigma}_l(t) &= -\gamma_l\sigma_l(t) - \alpha_l S_l(t) + \beta_l H,\\
\dot{\sigma}_r(t) &= -\gamma_r\sigma_r(t) - \alpha_r S_r(t) + \beta_r H
\end{aligned} \tag{1}$$

With arterial pulmonary pressure given by,

$$P_{ap}(t) = \frac{1}{c_{ap}}(V_0 - c_{as}P_{as}(t) - c_{vs}P_{vs}(t) - c_{vp}P_{vp}(t)), \tag{2}$$

Systemic and pulmonary blood flows given by,

$$\begin{aligned}
F_s(t) &= \frac{P_{as}(t) - P_{vs}(t)}{R_s},\\
F_p(t) &= \frac{P_{ap}(t) - P_{vp}(t)}{R_p}
\end{aligned} \tag{3}$$

If R_s and R_p are treated as constant parameters, we are modelling the resistance vessels as rigid tubes. Alternatively, during steady state we may model resistances by,

$$\begin{aligned}
R_p &= 1.965 + 1.17W,\\
R_s &= A_{pesk}C_{v_{O_2}},\\
A_{pesk} &= 177.3 + 1.17W
\end{aligned} \tag{4}$$

Cardiac outputs given are by,

$$\begin{aligned}
Q_l(t) &= H\frac{c_l P_{vp} f(S_l(t), P_{as}(t))(1 - e^{-\frac{t_d}{R_l c_l}})}{P_{as}(t)(1 - e^{-\frac{t_d}{R_l c_l}}) + f(S_l(t), P_{as}(t))e^{-\frac{t_d}{R_l c_l}}},\\
Q_r(t) &= H\frac{c_r P_{vs} f(S_r(t), P_{ap}(t))(1 - e^{-\frac{t_d}{R_r c_r}})}{P_{ap}(t)(1 - e^{-\frac{t_d}{R_r c_r}}) + f(S_r(t), P_{ap}(t))e^{-\frac{t_d}{R_r c_r}}},
\end{aligned} \tag{5}$$

with

$$f(s,p) = 0.5(s+p) - 0.5((p-s)^2 + 0.01)^{1/2}, \tag{6}$$

Diastole duration given by,

$$t_d = \left(\frac{60}{H}\right)^{\frac{1}{2}} \left(\left(\frac{60}{H}\right)^{\frac{1}{2}} - \kappa\right), \tag{7}$$

Where, from experimental results, heart rate is given by,

$$H = 35 MR_{O_2} + 66, \tag{8}$$

The metabolic oxygen demand is given by,

$$MRO_2(t) = MR^e_{O_2} + K\dot{C}_{v_{O_2}}(t), \tag{9}$$

with

$$MR^e_{O_2} = MR^r_{O_2} + \rho W, \tag{10}$$

and metabolic carbon dioxide production given by,

$$MR_{CO_2}(t) = RQ MR_{O_2}(t), \tag{11}$$

Finally,

$$\begin{aligned} C_{a_{O_2}}(t) &= K_1(1 - e^{-K_2 P_{a_{O_2}}}(t))^2, \\ C_{a_{CO_2}}(t) &= K_{CO_2} P_{a_{CO_2}}(t) + k_{CO_2}. \end{aligned} \tag{12}$$

Model Parameters

Parameter	Value
K_{CO_2}	0.244
k_{CO_2}	0.0065
K	19.2
K_1	0.2
K_2	0.05
$P_{I_{CO_2}}$	0
$P_{I_{O_2}}$	150
R_p	1.965
RQ	0.86
R_s	21.64
$V_{A_{CO_2}}$	3.0
$V_{A_{O_2}}$	2.5
$V_{T_{CO_2}}$	15
$V_{T_{O_2}}$	6
α_l	89.47
α_r	28.46
β_l	73.41
β_r	1.780

Parameter	Value
γ_l	37.33
γ_r	11.88
c_{as}	0.01002
c_{vs}	0.643
c_{ap}	0.03557
c_{vp}	0.1394
R_l	11.35
R_r	4.158
c_l	0.01289
c_r	0.06077
rho	0.011
V_0	5.0
MR_{O_2}	0.350
τ_p	0.5
κ	0.4
G_p	0.504
G_c	0.025
I_p	35.5
I_c	35.5

Table A: Parameter values

A model for ventilatory dead-space - model parameters

Patients	$\dot{V}_{D_B}$	a
CF01	5.6917	0.0391
CF02	2.8548	0.0986
CF03	2.6404	0.0891
CF04	2.1808	0.1500
CF05	0.8075	0.0876
CF06	2.4948	0.0339
CF07	1.3923	0.1850
CF08	1.9317	0.0535
CF09	0.9369	0.1467
CF10	1.9203	0.0493
CF11	2.2408	0.1455
CF12	1.7298	0.0498
CF13	3.9371	0.0649
CF14	2.3089	0.0889
CF15	3.7070	0.0658

Table B: Calculated values of $\dot{V}_{D_B}$ and a for the linear model of ventilatory deadspace.

Notational Defintions

Symbol	Meaning
a	Slope of the relationship between $\dot{V}_D$ and $W(t)$.
α_l	Coefficient of S_l in the equation for $\dot{\sigma}_l$.
α_r	Coefficient of S_r in the equation for $\dot{\sigma}_r$.
A_{pesk}	$R_s = A_{pesk} C_{v_{O_2}}$.
β_l	Coefficient of H in the equation for $\dot{\sigma}_l$.
β_r	Coefficient of H in the equation for $\dot{\sigma}_r$.
c	Scaling factor for converting between STPD (used for measuring blood gas concentrations) and BTPS (used for measuring blood volumes), $c = \frac{863}{P_a - 47}$.
c_{ap}	Compliance of the arterial section of the pulmonary system.
c_{as}	Compliance of the arterial section of the systemtic system.
c_{vp}	Compliance of the venous section of the pulmonary system.
c_{vs}	Compliance of the venous section of the systemic system.
c_l	Compliance of the relaxed left ventricle.
c_r	Compliance of the relaxed right ventricle.
$C_{a_{CO_2}}$	Concentration of CO_2 (both bound and dissolved) in the arterial blood.
$C_{a_{O_2}}$	Concentration of O_2 (both bound and dissolved) in the arterial blood.
$C_{T_{CO_2}}$	Concentration of CO_2 (both bound and dissolved) in the body tissue.
$C_{T_{O_2}}$	Concentration of O_2 (both bound and dissolved) in the body tissue.

$C_{v_{CO_2}}$	Concentration of CO_2 (both bound and dissolved) in the venous blood.
$C_{v_{O_2}}$	Concentration of O_2 (both bound and dissolved) in the venous blood.
$F_{A_{CO_2}}$	Fractional concentration of CO_2 in alveolar gas.
$F_{A_{O_2}}$	Fractional concentration of O_2 in alveolar gas.
$F_{I_{CO_2}}$	Fractional concentration of CO_2 in inspired gas.
$F_{I_{O_2}}$	Fractional concentration of O_2 in inspired gas.
F_p	Blood flow perfusing the lungs (pulmonary circuit).
F_s	Blood flow perfusing the tissue (arterial circuit).
H	Heart Rate.
G_c	Central controller gain factor (ventilation).
G_p	Peripheral controller gain factor (ventilation).
γ_l	Coeffecient of σ_l in the equation for $\dot{\sigma}_l$.
γ_r	Coeffecient of σ_r in the equation for $\dot{\sigma}_r$.
I_c	Central drive constant (ventilation).
I_p	Peripheral drive constant (ventilation).
K_1	O_2 dissociation curve constant.
K_2	O_2 dissociation curve constant.
K_{CO_2}	CO_2 dissociation curve constant.
k_{CO_2}	CO_2 dissociation curve constant.
κ	Duration of systole.
MR_{CO_2}	Metabolic rate of CO_2 production in the tissues.
MR_{O_2}	Metabolic rate of O_2 consumption in the tissues..
$P_{I_{CO_2}}$	Parital pressue of CO_2 in inspired air.
$P_{I_{O_2}}$	Parital pressue of O_2 in inspired air.
P_a	Ambient pressure.
$P_{a_{CO_2}}$	Parital pressue of CO_2 in arterial blood.
$P_{a_{O_2}}$	Parital pressue of O_2 in arterial blood.
$P_{A_{CO_2}}$	Parital pressue of CO_2 in alveolar blood.
$P_{A_{O_2}}$	Parital pressue of O_2 in alveolarl blood.
P_{ap}	Mean blood pressue in arterial section of the pulmonary system.
P_{as}	Mean blood pressue in arterial section of the systemic system.
P_{vp}	Mean blood pressue in venous section of the pulmonary system.
P_{vs}	Mean blood pressue in venous section of the systemic system.
$P_{v_{CO_2}}$	Parital pressue of CO_2 in venous blood.
$P_{v_{O_2}}$	Parital pressue of O_2 in venous blood.
Q_l	Left cardiac output.
Q_r	Right cardiac output.
R_p	Resistance of the pulmonary circuit.
R_l	Resistance of the left ventricle.
R_r	Resistance of the right ventricle.
RQ	$MR_{CO_2} = RQMR_{O_2}$.
R_s	Resistance of the systemic circuit.

ρ	Constant of W in the equation for MR_{O_2}.
S_l	Contractility (strength) of left ventricle.
S_r	Contractility (strength) of left ventricle.
σ_l	Derivative of $S_l(t)$.
σ_r	Derivative of $S_r(t)$.
t_d	Duration of diastole.
V_A	Alveolar gas volume.
V_D	Dead space volume.
V_{D_B}	Base dead space volume in linear equation for dead space ventilation.
V_T	Tidal volume.
$\dot{V}_A$	Alveolar ventilation.
$\dot{V}_D$	Dead space ventilations.
$\dot{V}_E$	Total ventilation.
V_{ap}	Volume of blood in pulmonary arterial compartment.
V_{as}	Volume of blood in systemic arterial compartment.
V_{vp}	Volume of blood in pulmonary venous compartment.
V_{vs}	Volume of blood in systemic venous compartment.
$V_{A_{CO_2}}$	Storage volume of CO_2 in the aveolar.
$V_{A_{O_2}}$	Storage volume of O_2 in the aveolar.
V_0	Total volume of blood.
W	Workload.

Table C: Definitions of model parameters

References

[1] Timischl S. (1998).
A Global Model for the Cardiovascular and Respiratory System: *Karl-Franzens-Universität Graz, Austria*

[2] A.G. Thin, J.D. Dodd, C.G. Gallagher, M.X. Firzgerald, P. Mclougjlin (2004).
Effect of respiratory rate on airway deadspace ventilation during exercise in cystic fibrosis *Respiratory Medicine*, **98**, 1063-1070.
